# Comparative Evaluation of the Effects of Adenotonsillar Hypertrophy on Oral Health in Children

**DOI:** 10.1155/2021/5550267

**Published:** 2021-04-02

**Authors:** Nilsu İnönü-Sakallı, Cemal Sakallı, Özgür Tosun, Damla Akşit-Bıçak

**Affiliations:** ^1^Department of Pediatric Dentistry, Near East University Faculty of Dentistry, Nicosia/ TRNC, 99138 Mersin 10, Turkey; ^2^Department of Otorhinolaryngology, Near East University Training and Research Hospital, Nicosia/ TRNC, 99138 Mersin 10, Turkey; ^3^Department of Biostatistics, Near East University Faculty of Medicine, Near East University, Nicosia/ TRNC, 99138 Mersin 10, Turkey

## Abstract

We aimed to investigate the oral health of children in terms of the presence of dental caries, periodontal health, halitosis, and dentofacial changes in patients who had adenotonsillar hypertrophy related to mouth breathing and compared these findings with nasal breathing healthy and adenotonsillectomy-operated children. The patient group comprised 40 mouth-breathing children who were diagnosed with adenotonsillar hypertrophy, while the control group consisted of 40 nasal breathing children who had no adenotonsillar hypertrophy. Forty children who had undergone an adenotonsillectomy operation at least 1 year prior to the study were included in the treatment group. Oral examinations of all children were conducted, and the parents were asked about medical and dental anamnesis, demographic parameters, toothbrushing and nutrition habits, oral health-related quality of life (OHRQoL), and symptoms of their children. Demographic parameters, toothbrushing and nutrition habits, and the presence of bad oral habits did not differ between groups (*p* > 0.05). Adenotonsillectomy is associated with a remarkable improvement in symptoms; however, some symptoms persist in a small number of children. The salivary flow rate, dmft/s, DMFT/S index, plaque, and gingival index scores did not differ between groups (*p* > 0.05). The patient group showed higher rates of halitosis when compared with the treatment and control groups (*p* < 0.001). Mouth breathing due to adenotonsillar hypertrophy caused various dentofacial changes and an increase in Class II division 1 malocclusion (*p* < 0.001). It was shown that adenotonsillar hypertrophy does not negatively affect OHRQoL, it could be a risk factor for dental caries, periodontal diseases, and halitosis, but by ensuring adequate oral health care, it is possible to maintain oral health in children with adenotonsillar hypertrophy. Also, it is recommended that orthodontic treatment should start as soon as possible if it is required. In this context, otorhinolaryngologists, pedodontists, and orthodontists should work as a team in the treatment of children with adenotonsillar hypertrophy.

## 1. Introduction

The Waldeyer's ring is known as the lymphoid tissue surrounding the opening of the oral and nasal cavities to the pharynx and consists of the palatine, pharyngeal (adenoids), lingual, and tubal tonsils together with lymphatic tissue scattered along the mucosal lining of the pharynx. It serves as the first-line defence mechanism against microorganisms and antigenic substances [1, 2]. Adenoid and tonsil tissues begin to grow in the first years of life with gradual growth up to the age of five and have the feature of regressing and diminishing in size from childhood to adolescence [2]. Hence, adenotonsillar hypertrophy is defined as the condition in which adenotonsillar tissue enlarges in dimension and accordingly invades a larger space in the nasopharynx wall, which can lead to the development of serious problems such as snoring, abnormal and sleep-disordered breathing, obstructive sleep apnoea, speaking, smelling, tasting, swallowing difficulties, mouth breathing, and orofacial problems [3–7].

A compensatory mouth breathing habit among young children is widely accepted as one of the primary outcomes of the mechanical obstruction of the airflow passage because of adenotonsillar hypertrophy together with untreated allergic rhinitis [8, 9]. Mouth breathing due to adenotonsillar hypertrophy has been shown to cause the majority of dentofacial changes such as V-shaped narrowing in the maxillary arch, opening of the lips and positioning of the tongue below its normal position, retrognathic mandible, increased overjet, anterior and posterior crossbite, and anterior open bite. All these changes affecting the dentofacial development are described as “Adenoid face” [8, 10–12]. In order to treat chronic upper airway obstruction in the presence of adenotonsillar hypertrophy, adenoidectomy or adenotonsillectomy surgeries are generally conducted on children and it has been reported that most dentofacial anomalies are reversible within the first year after adenoidectomy [13, 14]. Furthermore, mouth breathing due to adenotonsillar hypertrophy has been shown to induce the formation of dental caries, halitosis, and periodontal problems because of the decrease in the cleaning effect of saliva and the open positioning of the lips [11, 12, 15]. However, conflicting results can be found in the literature on this topic, and the effect of adenotonsillectomy treatment on the improvement of halitosis, periodontal problems, dental caries formation, and oral health-related quality of life (OHRQoL) has not been sufficiently clarified. Thus, according to the above information, the purpose of this study was to evaluate the oral health of children in terms of the presence of dental caries, periodontal health, halitosis, and malocclusions in children who have adenotonsillar hypertrophy related mouth breathing and to compare these findings with healthy children with nasal breathing and children who have undergone adenotonsillectomy treatment. Our study is based on the hypothesis that mouth breathing due to adenotonsillar hypertrophy may negatively affect OHRQoL and cause an increase in malocclusion, halitosis, dental caries, and periodontal diseases in children when compared with healthy controls and adenotonsillectomy operated children. Evaluating the oral health status of children who have undergone adenotonsillectomy operations provided us with the opportunity to examine the benefits of adenotonsillectomy surgery on oral health and thus enabling us to make a significant contribution to this field, which needs support in the literature.

## 2. Materials and Methods

### 2.1. Patient Recruitment

Eighty children aged 3-14 who presented to the Near East University Hospital Otorhinolaryngology Clinic due to ear, nose, and throat (ENT) complaints and consented to participate in the study constituted the patient and control groups. After adenoid and tonsil evaluation, 40 children who were diagnosed with adenotonsillar hypertrophy together with mouth breathing represented the patient group and 40 children who had no adenotonsillar hypertrophy together with nasal breathing formed the control group of this study. Also, 40 children who had undergone an adenotonsillectomy operation at the Near East University Hospital Otorhinolaryngology Clinic at least 1 year prior to the study were included in the treatment group. Treatment group patients were contacted by phone using their contact details from the patient records and were requested to visit the Near East University, Faculty of Dentistry, Paediatric Dentistry Department for oral examination.

G∗ Power (Version 3.1.9.4) for Mac software was used for sample size calculation. Based on similar studies [16, 17] in the literature and as a result of the statistical evaluation with 80% statistical power and 5% margin of error, it was found that 35 patients should be included in each group. Considering the possible errors that might occur during the study resulting in patient or data loss, the decision was made to incorporate 40 patients in each group. Thus, a total of 120 children were enrolled in the study. This study was conducted between February 2020 and November 2020.

### 2.2. Ethical Approval

Ethical approval was obtained from Near East University Clinical Research Ethics Committee (NEU/2019/71-882). Parents of all 120 children who accepted to participate in this study read and signed written informed consent forms about the study.

### 2.3. Inclusion Criteria

The patient group included children with adenotonsillar hypertrophy and mouth breathing, between the ages of 3-14, no chronic or systemic disease, no immune system deficiency, and the presence of adenotonsillar hypertrophy with surgical indications for adenotonsillectomy together with mouth breathing. The control group included children without adenotonsillar hypertrophy and nasal breathing, between the ages of 3-14, no chronic or systemic disease, no immune system deficiency, no history of adenotonsillar hypertrophy or previous adenotonsillectomy surgery, and no history of mouth breathing and children with nasal breathing. The treatment group included children who had undergone adenotonsillectomy surgery between the ages of 3-14, no chronic or systemic disease, no immune system deficiency, and children who had undergone adenotonsillectomy surgery at least 1 year prior to the study.

### 2.4. Exclusion Criteria

For all groups, exclusion criteria included previous facial surgery, dysmorphic or craniofacial syndromes, septum deviation, chronic disease, upper respiratory tract obstruction other than adenoid vegetation, acute upper respiratory tract infection period, finger sucking habits, previous orthodontic treatment, and ongoing orthodontic treatment [18].

### 2.5. Medical and Dental Anamnesis

The demographic information together with dental and medical anamnesis of a total of 120 children were obtained in detail and recorded in anamnesis forms. Questions about the parents' income level, education level, children's previous dental treatment experience, presence of snoring, open mouth during sleep, daytime somnolence, restless sleep, salivate on the pillow during sleeping, thirsty awakening at nights, dry mouth, bedwetting, headache, aggression, lack of attention, hyperactivity, abnormal breathing, difficulty in swallowing, smelling, tasting and speaking difficulties, negative effect on school performance, and the presence of allergies were asked to the parents, and their responses were recorded on the anamnesis forms. In addition, parents were asked about the frequency of their child's toothbrushing, their participation in toothbrushing, nutrition habits of the child, fluoride concentration of the child's toothpaste, the presence of bad oral habits such as bruxism, nail biting, lip biting, tongue-thrusting, lip sucking, and if they detected bad breath in their child or themselves.

### 2.6. Ear Nose Throat Examination

Eighty children who presented to the Near East University Hospital Otorhinolaryngology clinic underwent examination by the same otolaryngologist. Flexible fiberoptic nasopharyngolaryngoscopy (FNFL) was used for direct visualization of the adenoid and tonsils [18, 19]. Palatine tonsil hypertrophy was determined according to the Brodsky and Koch criteria [20]. Patients with adenoid hypertrophy were evaluated with FNFL in two categories according to the degree of nasopharyngeal obstruction (<75% and ≥75%) [21]. Children with Grade 4 and ≥75 obstruction of the airway were included in the study as the patient group. Children without adenotonsillar hypertrophy were included in the study as the control group.

### 2.7. Oral Examination

The oral examinations of all 120 children were performed by one examiner at the Near East University, Faculty of Dentistry, Paediatric Dentistry Department. Patients were requested to sit in a dental chair. Under dental chair light, the surfaces of the teeth were investigated by using a dental mirror and dental explorer after drying with air. The dentition period for each child was recorded as primary, permanent, and mixed dentition. In addition, the DMFT/S values for permanent teeth and dmft/s values for primary teeth (D,d: decayed, M,m: missing, F,f: filled, and T,t: total) were detected consistent with World Health Organization standards [22]. Plaque index and gingival index values of children were evaluated according to Silness and Löe [23] and Löe and Silness [24], respectively. Unstimulated salivary flow rates were assessed by asking the children to spit into a 15 ml falcon tube placed in a funnel for 5 minutes and were calculated as millilitres per minute (ml/min). Children were instructed not to swallow during saliva collection [25]. In the evaluation of halitosis, the organoleptic measurement technique was used. Children were asked to hold a plastic tube in their mouth and told to slowly exhalate into the tube. During exhalation, the examiner evaluated the odor from the other side of the tube. The organoleptic measurement of odor was classified as stated in a recent study [26].

In the evaluation of malocclusion, the presence of a V-shaped narrowing in the maxillary arch, adenoid face, teeth crowding, macroglossia, open mouth posture, anterior and lower tongue position, mandibula (normal, prognathic, and retrognathic), position of the maxillary and mandibular anterior teeth, and presence of dry, chapped lips were assessed. In addition, vertical, transversal, and sagittal interarch dental occlusion were evaluated [21]. Vertical relationship was categorized as normal, posterior open bite, anterior open bite, and deep bite. Transversal relationship was categorized as normal and posterior crossbite. Sagittal relationship was categorized as normal occlusion, Class I malocclusion, Class II Division 1 malocclusion, Class II Division 2 malocclusion, and Class III malocclusion [21].

### 2.8. Oral Health-Related Quality of Life (OHRQoL) Assessment

The Turkish version of the Early Childhood Oral Health Impact Scale (ECOHIS) was used to assess the OHRQoL of children who participated in this study, which was shown to have acceptable validity and reliability by Peker et al. [27]. Parents of all children rated 13 questions in total. The first part is the child impact section and includes 4 domains, namely, child symptoms, functions, psychology, self-image, and social interaction. The second part is the parent impact section and includes two domains, namely, parental distress and family function. A five-point Likert scale was used for rating each item (0 = never; 1 = hardly ever; 2 = occasionally; 3 = often; 4 = very often; and 5 = do not know). Higher scores indicated more problems and/or greater impacts [27].

### 2.9. Statistical Analysis

For all study variables, descriptive statistics were calculated. Qualitative variables were described with frequency and percentage, while arithmetic mean, standard deviation (SD), and standard error of the mean (SE) were calculated for quantitative variables. Pearson Chi-Square test, Fisher's Exact test, and trend test for ordinal variables were used to compare the distribution differences of qualitative variables between study groups. Following the Shapiro-Wilk test of normality, the nonparametric Kruskal Wallis test was applied to compare the three study groups with respect to the quantitative variables. The level of significance was accepted as 0.05. All calculations and statistical analyses were carried out with Jamovi (Version 1.2.27.0 for Mac) software.

## 3. Results

A total of 120 children participated in this study. The mean ages of the groups were 7.35 ± 3.55 for the patient group, 8.08 ± 2.89 for the treatment group, and 8.08 ± 2.89 for the control group. No statistically significant difference was found in the mean ages of the study groups (*p* = 0.284). Of the children participating in the study, 53 (44.2%) were girls and 67 (55.8%) were boys, and no difference was found between the groups in terms of gender distribution (*p* = 0.9671). The children included in this study were selected in such a manner that there was an equal distribution between the groups in terms of age, gender, and dentition period. Family income (*p* = 0.262) and parental education level (*p* = 0.098) also did not differ between groups. It was detected that 57.5% of children in the patient group had never visited a dentist, but 82.5% of children in the control group had visited a dentist before (Table 1).

Table 2 displays the symptoms of the patient and treatment groups. No statistically significant differences were found in terms of tasting (*p* = 0.077), smelling (*p* = 0.288), speaking (*p* = 0.251) difficulties, hyperactivity (*p* = 0.822), lack of attention (*p* = 0.371), aggression (*p* = 0.366), headache (*p* = 0.456), bedwetting (*p* = 0.152), and negative affection of school performance (*p* = 0.235) among the patient and treatment groups. It was found that in the patient group, 21 (52.5%) children had difficulty in swallowing, 33 (82.5%) children had abnormal breathing, 36 (90.0%) had dry mouth, 36 (90.0%) had thirsty awakening at nights, 35 (87.5%) had salivate on the pillow during sleeping, 30 (75.0%) had restless sleep, 15 (37.5%) had daytime somnolence, and 37 (92.5%) had open mouth during sleeping. All children in the patient group had snoring and mouth breathing. These findings were found to be significantly higher when compared with the findings of the treatment group (*p* < 0.001).

Table 3 illustrates the toothbrushing and nutrition habits of all children in this study. Most of the children performed toothbrushing at least once a day and used fluoridated toothpaste; however, it was detected that parents did not brush their child's teeth and performed supervised toothbrushing. No statistically significant differences were detected in the nutrition habits of children when the different groups were compared. Only candy and lollipop consumption frequency differed as 31 (77.5%) children in the treatment group consumed candy and lollipops less than once a week, followed by 26 (65%) children in the control and 18 (45%) children in the patient group (*p* = 0.010).

The salivary flow rate, dmft/s, DMFT/S index, and plaque and gingival index scores did not differ between the patient, treatment, and control groups (*p* > 0.05) (Table 4). A total of 33 children in the treatment group (82.5%), 24 children in the patient group (60%), and 24 in the control group (60%) had dental plaque on the buccal surface of the anterior teeth, and a statistically significant difference was found between groups (*p* = 0.046).

Table 5 displays the statistically significant difference in the organoleptic assessment of halitosis among the study groups (*p* < 0.001). Twenty-seven (67.5%) children demonstrated weak but clearly noticeable odor, and 13 (32.5%) children had moderate or strong odor in the patient group. However, 21 children in the treatment group (52.5%) and 24 in the control group (60%) had no or barely noticeable odor (*p* < 0.001). In addition, the parents of 39 (97.5%) children in the patient group perceived odor, whereas 19 (47.5%) and 14 (35.0%) parents detected such odors in the treatment and control groups, respectively.

The presence of bad oral habits did not differ between patient, treatment, and control groups (*p* > 0.05) (Table 6). It was determined that 32 (26.7%) children were in primary, 68 (56.7%) children were in mixed, and 20 (16.7%) children were in permanent dentition. The presence of teeth crowding did not differ between groups (*p* = 0.051). It was detected that 38 (95%) children in the patient group, 38 (95.0%) children in the treatment group, and none of the children in the control group showed V-shaped narrowing in their maxillary arch (*p* < 0.001). Adenoid face was detected in 38 (95%) children in the patient group, 30 (75%) children in the treatment group, and none of the children in the control group (*p* < 0.001). Macroglossia was detected in 27 (67.5%) children in the treatment group, 26 (65.0%) children in the patient group, and 5 (12.5%) children in the control group (*p* < 0.001). Open mouth posture was found in 33 (82.5%), 27 (67.5%), and 4 (10.0%) children in the patient, treatment, and control groups, respectively. It was found that 26 (65%) children in the patient group, 25 (62.5%) children in the treatment group, and none of the children in the control group had anterior or lower tongue positions. Thirty-seven (92.5%) children had dry, chapped lips in the patient group; however, this finding was detected in only 7 (17.5%) children in the treatment group and 1 (2.5%) child in the control group (Table 7).

Mandibula position was mainly (92.5%) retrognathic among the patient group followed by children in the treatment group (75.0%). However, in the control group, it was found that the mandibular position was predominantly (87.5%) normal. Prominent position of the maxillary anterior teeth was detected in 15 (37.5%), 18 (45%), and 3 (7.5%) children in the patient, treatment, and control groups, respectively. Also, retrognathic position of the mandibular anterior teeth was detected in 20 (50.0%) children in the patient group, 10 (25.0%) children in the treatment group, and 1 (2.5%) child in the control group (Table 7).

Table 8 displays the results of the interarch dental occlusal assessment of the study participants. Class II Division 1 malocclusion was detected in 18 (64.3%) children in the patient group, 15 (50.0%) children in the treatment group, and in only 4 (11.8%) children in the control group. A statistically significant difference was found among the study groups (*p* < 0.001). Class I malocclusion was detected in 4 (14.3%), 6 (20.0%), and 10 (29.4%) children in the patient, treatment, and control groups, respectively. Normal occlusion was detected in 20 (58.8%) children in the control group, 9 (30%) children in the treatment group, and only 6 (21.4%) children in the patient group (*p* < 0.001). No statistically significant differences were detected in the frequency of the presence of posterior crossbite (*p* = 0.335), anterior open bite, and deep bite (*p* = 0.244) between groups.

Table 9 shows the OHRQoL of all children who participated in the study. When the ECOHIS scores were compared between the groups, difference was only found between the children's self-image and social interaction scores (*p* = 0.049). This difference was between the patient and control groups (*p* = 0.012). No difference was found in terms of the self-image and social interaction scores of the children between the patient and treatment groups (*p* = 0.170).

## 4. Discussion

Adenotonsillar hypertrophy (AH) is regarded as a prevalent disease among children and one of the primary causes of upper respiratory obstruction [28, 29]. In this study, it was found that children with adenotonsillar hypertrophy experienced various symptoms that were reported in the literature [8, 10, 30]. Symptoms that were detected in higher rates in the patient group when compared with children in the treatment group were mouth breathing, followed by snoring, open mouth during sleep, dry mouth, thirsty awakening at nights, salivate on the pillow during sleeping, abnormal breathing, and restless sleep. The lower rates of symptoms detected in the treatment group showed that adenotonsillectomy surgery has a positive effect on relieving such symptoms. Elsherif and Kareemullah [30] conducted a study involving 76 children aged between 3 and 12 with large adenoids and tonsils who had surgery in order to improve upper airway obstruction. The researchers reported that after surgery, nearly all patients demonstrated recovery of all symptoms such as snoring, mouth breathing, sleep apnoea, and daytime somnolence. In accordance with this study, Elsherif and Kareemullah [30] reported that children who had adenotonsillar hypertrophy showed significant improvement after adenotonsillectomy surgery. Orji and Ezeanolue [13] conducted a study on 59 children with chronic upper airway obstruction and examined their improvements in breathing difficulties and sleep disturbances following adenotonsillectomy surgery. The researchers reported that the most improved symptom was snoring, and the least was daytime somnolence. In our study, the symptom with the most significant improvement was mouth breathing followed by snoring, and the least improvement was observed in hyperactivity. Orji and Ezeanolue [13] reported that although they found significant recovery in obstructive symptoms in most of the children after adenotonsillectomy, some children continued mouth breathing and snoring as with our findings. Several children in the treatment group continued snoring and mouth breathing along with other symptoms in this study. According to our findings, we concluded that although adenotonsillectomy is associated with remarkable improvement in the symptoms of children with adenotonsillar hypertrophy, some symptoms persist in a small number of children.

Patients with adenotonsillar hypertrophy mainly breath through the mouth [8, 9]. In mouth breathing patients, there may be a decrease in the amount of saliva through evaporation. Saliva has many important functions in maintaining oral health. These include self-cleaning of the oral cavity, buffering of the acids, antimicrobial properties, and remineralization of demineralized enamel. A reduction in resting salivary flow is related to increased numbers of candida and lactobacilli species, decreased plaque pH, and a higher risk of caries [31]. Ahmed [17] reported that children with chronic tonsillitis had higher mean values of dental caries when compared to children with intact tonsils. The researchers stated that dental caries and peritonsillar infections possess the same microbial pathogens. Ballıkaya et al. [11] reported that mouth breathing might be related to the severity of dental caries. The results of the studies by Ahmet [17] and Ballıkaya et al. [11] contradict our findings because no significant relationship was found in the salivary flow rate, dmft/s, DMFT/S index scores between the patient, treatment, and control groups of this study. Yıldırım and Aktören [32] conducted a study among 75 mouth-breathing children and 25 nasal breathing children aged between 5 and 12 years, and no statistically significant difference was found between the df, dfs, DMF, DMFS values of the mouth and nasal breathing children. Mummolo et al. [33] also reported that salivary flow rates did not differ between 20 mouth-breathing patients and the same number of nasal breathing patients. Alqutami et al. [34] investigated the effect of mouth breathing on the prevalence of dental caries but they were unable to show such a relationship. Weiler et al. [35] conducted a study among 61 adolescents aged 10-19 years, where 30 were mouth breathers and 31 were nose breathers. The researchers reported no difference in salivary flow rates among the nasal and mouth breathing patients. Also, they did not find any difference between the DMFT scores in the two groups. Koga-Ito et al. [15] conducted a study among 30 mouth-breathing and healthy children and found no difference in terms of salivary flow rate among the study and control groups. The findings of the present study were found to be in accordance with the findings of Yıldırım and Aktören [32], Mummalo et al. [33], Alqutami et al. [34], Weiler et al. [35], and Koga-Ito et al. [15]. Dental caries is a multifactorial disease, and various factors play a role in the formation and progression of dental caries such as biological factors, the presence of cariogenic microorganisms, dietary habits, oral hygiene practices, parental education, and socioeconomic status [36]. Thus, in this study, these factors were also investigated. No statistically significant difference was found in the family income and parental education status among study groups, and both family income and education status were found to be high and satisfactory among the parents of all children. Furthermore, toothbrushing frequency, supervised toothbrushing, usage of fluoridated toothpaste, and nutritional habits did not differ among the study groups apart from the higher frequency of candy and lollipop consumption among the patient group compared with the treatment and control groups. Most of the children in all study groups said they brushed their teeth at least once a day and used fluoridated toothpaste. The similar results obtained with respect to those factors among the study groups could be the reason for the lack of difference in terms of dental caries formation. According to our findings, we could not demonstrate a relationship between adenotonsillar hypertrophy and dental caries formation. Furthermore, when the ECOHIS scores were compared between groups, there were no statistically significant differences found between groups in terms of child symptoms, function, psychology, family distress, and function. Therefore, it was shown that adenotonsillar hypertrophy did not contribute negatively to the OHRQoL of the children; it might be a risk factor for dental caries, but by ensuring adequate oral health care, it is possible to prevent the formation of dental caries in children with adenotonsillar hypertrophy.

Mouth breathing is also regarded as one of the factors that contribute to the initiation and progression of periodontal diseases. Chronic mouth breathing-related anterior open bite is linked with a high incidence of periodontal problems. Possible causes include the lack of cleansing effect of saliva, reduced epithelial defence to dental plaque, and dehydration of the gingival surface [31, 37]. Yıldırım and Aktören [32] found no significant differences in terms of marginal gingivitis between nose and mouth breathing children. In addition, plaque indices were found to be higher in nose breathing children compared to mouth breathing children. Alqutami et al. [34] also failed to demonstrate a relationship between the mouth breathing habit and the prevalence of gingivitis based on the examination of mandibular left central incisor and maxillary right central incisor. The plaque and gingival index evaluation findings of this study were in line with the findings of Yıldırım and Aktören [32] and Alqutami et al. [34]. On the other hand, the results of this study were not in agreement with those of Gulati et al. [38], Mummolo et al. [33], Demir et al. [16], and Nascimento Filho et al. [37]. Gulati et al. [38] reported that because of insufficient lip height in mouth breathing patients, plaque accumulation is high in the anterior region, so gingival index values are higher. Mummolo et al. [33] reported that plaque index values were higher in 20 mouth-breathing patients when compared with the same number of nasal breathing patients. Demir et al. [16] investigated 20 patients with adenotonsillar hypertrophy and 15 children without adenotonsillar hypertrophy and concluded that adenotonsillar hypertrophy leads to the occurrence of gingival disease and plaque accumulation and improves gingival health after surgery. In our study, plaque and gingival index scores did not differ between patient, treatment, and control groups. However, the incidence of dental plaque on the buccal surface of the anterior teeth was found to be higher than the posterior teeth among all study participants. The reason for this result might be because even though open mouth posture was detected in higher rates among patient and treatment groups when compared with the control group, the salivary flow rates did not differ between groups, most of the children perform mechanical plaque removal at least once a day, and only few children exhibited anterior open bite.

Pathogenesis of adenoid hypertrophy is one of the possible causes of halitosis [39]. In the formation of halitosis, mouth breathing is also important in addition to plaque accumulation, presence of dental caries, and tooth brushing habits [40]. The organoleptic test is accepted as the gold standard in oral malodor diagnosis, which can detect the strength of smell exhalated through the mouth or the nose [39]. Thus, in this study, the organoleptic measurement technique was used in the evaluation of halitosis. Dinc et al. [41] reported a statistically significant relationship between halitosis and adenoid hypertrophy and valuable recovery in halitosis after adenoidectomy surgery. Aw et al. [42] reported that adenotonsillar hypertrophy and tonsillitis are the main throat disorders that cause halitosis. Motta et al. [43] and Alqutami et al. [34] also demonstrated a statistically significant relationship between mouth breathing and halitosis. Yıldırım and Aktören [32] found halitosis in 58.7% of mouth breathing children and 64% of children with normal breathing, but they could not show a difference in terms of halitosis between the studied groups. In this study, the patient group showed higher rates of halitosis when compared with the treatment and control groups. The findings of the present study were found to be accordance with Dinc et al. [41], Aw et al. [42], Motta et al. [43], and Alqutami et al. [34], but not in accordance with Yıldırım and Aktören [32]. Adenoid hypertrophy can occur because of infectious and noninfectious etiologies [44]. Tonsillitis is one of the reasons for oral malodor in healthy individuals [26]. Parapharyngeal infections and nasal secretions might be the reasons for the higher rates of halitosis detected in the patient group when compared with the treatment and control groups of this study.

Morais-Almeida et al. [9] reported that mouth breathing is frequently associated with bad oral habits; however, the results obtained in the present study failed to demonstrate such a relationship as the presence of nail biting, lip biting, lip sucking, tongue thrusting, and bruxism did not differ between the patient, treatment, and control groups. Although the prevalence of children with nail biting and bruxism was slightly higher in the patient group, no statistically significant differences were found.

Mouth breathing due to adenotonsillar hypertrophy has been shown to cause most dentofacial changes [2, 8, 10–12, 45], and it has been reported that the majority of dentofacial anomalies are reversible within the first year after an adenoidectomy [13, 14]. In our study, in accordance with the literature [2, 8, 10–12, 45], we found that adenotonsillar hypertrophy caused dentofacial changes. A V-shaped narrowing in the maxillary arch and the presence of adenoid face were found to be statistically high in the patient group when compared with the control group, which concurs with the findings of Yıldırım and Aktoren [32]. Open mouth posture was observed in nearly 80% percent of children in the patient group in line with the findings of Yıldırım and Aktören [32], Lopatienė and Bbarskars [45], and Osiatuma et al. [46]. The statistically significant higher prevalence of anterior and lower tongue position detected in the patient group was similar to the findings of Osiatuma et al. [46], but the higher prevalence of anterior and lower tongue position and retrognathic position of the mandibula was detected in the patient group when compared with the control group not in line with the findings of Yıldırım and Aktören [32]. Furthermore, a prominent position of the maxillary anterior teeth was found in nearly 38% of children in the patient group, but only 8% percent of children in the control group. This finding was in accordance with Lopetiene and Bbarskars [45] and Bassheer et al. [47]. Statistically significant higher rates of Class II Div 1 malocclusion were detected in the patient group when compared with the control group. Similar observations were reported by Grippadue et al. [48], Lopatiene and Bbarskars [45], and Osiatuma et al. [46]. The presence of posterior crossbite among the patient group was slightly higher when compared with the control group. This finding was not found statistically significant, which is in line with Yıldırım and Aktören [32] and Melink et al. [49] but contradicts the findings of Grippaduo et al. [48] and Osiatuma et al. [46]. Furthermore, the prevalence of anterior open bite among the patient group was slightly higher in the patient group when compared with the control group, but the difference was not statistically significant. Thus, this finding was not in accordance with Grippaudo et al. [48], Yıldırım and Aktören [32], and De Lira et al. [8]. In addition, high and very similar rates of dentofacial changes were detected in the treatment group and patient groups. Thus, the high and very similar detection rates of V-shaped narrowing in the maxillary arch, adenoid face, macroglossia, open mouth posture, anterior and lower tongue position, retrognathic position of mandibula, prominent position of maxillary anterior teeth, retrognathic position of mandibular anterior teeth, and Class II division 1 malocclusion in the patient and treatment group compared to the control group indicated that adequate recovery could not be achieved on these findings after adenotonsillectomy surgery. Higher self-image and social interaction scores in the assessment of OHRQoL could originate from the dentofacial changes and halitosis among the patient group when compared with the control group. The lack of difference between the patient and treatment groups in terms of self-image might be because of the previously mentioned fact that adequate recovery of dentofacial changes was not achieved after adenotonsillectomy surgery. In accordance with our findings, Pereira et al. [50] reported that adenotonsillar hypertrophy is associated with mouth breathing and can cause facial changes, while adenotonsillectomy is not sufficient for improvement and recommended facial orthodontic treatments in order to achieve functional and morphological healing. William and Mahony [51] reported that by the age of 12, 80%-90% of craniofacial growth is complete, and the early prevention and management of young patients with increased nasal airway resistance have vital importance.

## 5. Conclusion

In conclusion, this study demonstrated that mouth breathing due to adenotonsillar hypertrophy may cause various dentofacial changes and an increase in Class II division 1 malocclusion and halitosis. However, we could not demonstrate such a relationship with dental caries and periodontal diseases. It was detected that adequate recovery could not be achieved on dentofacial changes after adenotonsillectomy surgery. In order to improve the dentofacial changes after surgery, it is recommended that orthodontic treatment should start as soon as possible if indicated. Furthermore, it is also extremely important to consult with a paediatric dentist in order to perform oral rehabilitation, determine risk factors, educate and support the child and family about oral health, and prevent the formation of new carious lesions by applying preventive measures and remineralisation agents. It was shown that adenotonsillar hypertrophy does not negatively affect OHRQoL, but it could be a risk factor for dental caries, periodontal diseases, and halitosis; however, by ensuring adequate oral health care, it is possible to maintain oral health in children with adenotonsillar hypertrophy. In this context, otorhinolaryngologists, pedodontists, and orthodontists should work as a team in the treatment of children with adenotonsillar hypertrophy.

## Figures and Tables

**Table 1:** Demographic parameters.

		Patient group (*N* = 40)	Treatment group (*N* = 40)	Control group (*N* = 40)	*p* value
Gender (*n*-%)	Girl	17 (42.5%)	18 (45.0%)	18 (45.0%)	0.967^1^
	Boy	23 (57.5%)	22 (55.0%)	22 (55.0%)	
Age	Mean	7.35	8.075	8.075	0.284^2^
	SD	3.5486	2.8946	2.8946	
	SE	0.56108	0.45768	0.45768	
Family income (*n*-%)	3.000–6.000 ₺	10 (25.0%)	3 (7.5%)	7 (17.5%)	0.262^1^
	6.000–9.000 ₺	15 (37.5%)	15 (37.5%)	13 (32.5%)	
	≥10.000 ₺	15 (37.5%)	22 (55.0%)	20 (50.0%)	
Parental education level (*n*-%)	Primary school	2 (5.0%)	1 (2.5%)	0 (0.0%)	0.098^3^
	Middle school	4 (10.0%)	1 (2.5%)	3 (7.5%)	
	High school	12 (30.0%)	6 (15.0%)	10 (25.0%)	
	University	17 (42.5%)	26 (65.0%)	20 (50.0%)	
	Master degree	5 (12.5%)	5 (12.5%)	5 (12.5%)	
	Doctorate degree	0 (0.0%)	1 (2.5%)	2 (5.0%)	
Previous dental visit (*n*-%)	Yes	17 (42.5%)	25 (62.5%)	33 (82.5%)	0.001^1^
	No	23 (57.5%)	15 (37.5%)	7 (17.5%)	

^1^Pearson's Chi-squared test, ^2^Kruskal Wallis Test, and ^3^Trend test for ordinal variables.

**Table 2:** Symptoms of patient and treatment groups.

	Patient group (*N* = 40)	Treatment group (*N* = 40)	*p* value
Tasting difficulty	3 (7.5%)	0 (0.0%)	0.077
Smelling difficulty	6 (15.0%)	3 (7.5%)	0.288
Speaking difficulty	18 (45.0%)	13 (32.5%)	0.251
Difficulty in swallowing	21 (52.5%)	4 (10.0%)	<0.001
Abnormal breathing	33 (82.5%)	2 (5.0%)	<0.001
Hyperactivity	23 (57.5%)	22 (55.0%)	0.822
Lack of attention	22 (55.0%)	18 (45.0%)	0.371
Aggression	19 (47.5%)	15 (37.5%)	0.366
Headache	5 (12.5%)	3 (7.5%)	0.456
Bedwetting	2 (5.0%)	0 (0.0%)	0.152
Dry mouth	36 (90.0%)	9 (22.5%)	<0.001
Thirsty awakening at nights	36 (90.0%)	5 (12.5%)	<0.001
Salivate on the pillow during sleeping	35 (87.5%)	7 (17.5%)	<0.001
Negative affection of school performance	5 (12.5%)	2 (5.0%)	0.235
Restless sleep	30 (75.0%)	3 (7.5%)	<0.001
Daytime somnolence	15 (37.5%)	2 (5.0%)	<0.001
Open mouth during sleeping	37 (92.5%)	15 (37.5%)	<0.001
Snoring	40 (100.0%)	4 (10.0%)	<0.001
Mouth breathing	40 (100.0%)	3 (7.5%)	<0.001

Pearson's Chi-squared test.

**Table 3:** Toothbrushing and nutrition habits.

		Patient group (*N* = 40)	Treatment group (*N* = 40)	Control group (*N* = 40)	*p* value
Toothbrushing frequency	At least once a day	36.0 (90.0%)	32.0 (80.0%)	33.0 (82.5%)	0.444
	Several times a week or never	4.0 (10.0%)	8.0 (20.0%)	7.0 (17.5%)	
Supervised toothbrushing	Generally	13.0 (32.5%)	11.0 (27.5%)	19.0 (47.5%)	0.152
	Occasionally or never	27.0 (67.5%)	29.0 (72.5%)	21.0 (52.5%)	
Used toothpaste	Nonfluoridated	1.0 (2.5%)	2.0 (5.0%)	5.0 (12.5%)	0.175
	Fluoridated	39.0 (97.5%)	38.0 (95.0%)	35.0 (87.5%)	
Nutrition habits	Milk and milk products				
	Less than once a week	4.0 (10.0%)	4.0 (10.0%)	7.0 (17.5%)	0.504
	At least once a day	36.0 (90.0%)	36.0 (90.0%)	33.0 (82.5%)	
	Fruits and vegetables				
	Less than once a week	1.0 (2.5%)	5.0 (12.5%)	1.0 (2.5%)	0.088
	At least once a day	39.0 (97.5%)	35.0 (87.5%)	39.0 (97.5%)	
	Carbonated beverages				
	Less than once a week	26.0 (65.0%)	30.0 (75.0%)	32.0 (80.0%)	0.303
	At least once a day	14.0 (35.0%)	10.0 (25.0%)	8.0 (20.0%)	
	Chocolate and biscuits				
	Less than once a week	7.0 (17.5%)	7.0 (17.5%)	8.0 (20.0%)	0.946
	At least once a day	33.0 (82.5%)	33.0 (82.5%)	32.0 (80.0%)	
	Chips and snacks				
	Less than once a week	18.0 (45.0%)	24.0 (60.0%)	26.0 (65.0%)	0.171
	At least once a day	22.0 (55.0%)	16.0 (40.0%)	14.0 (35.0%)	
	Candy and lollipops				
	Less than once a week	18.0 (45.0%)	31.0 (77.5%)	26.0 (65.0%)	0.010
	At least once a day	22.0 (55.0%)	9.0 (22.5%)	14.0 (35.0%)	

Pearson's Chi-squared test.

**Table 4:** Assessment of caries index, plaque index, and gingival index.

	Group	*N*	Mean	SD	SE	*p*
Salivary flow rate	Patient	40	0.73	0.5441	0.08602	0.377
	Treatment	40	0.6325	0.4548	0.07191	
	Control	40	0.7725	0.5435	0.08593	
dmft	Patient	33	2.51515	2.9593	0.51515	0.288
	Treatment	35	2.62857	2.8808	0.48695	
	Control	34	3.58824	3.1249	0.53591	
dmfs	Patient	33	3.69697	4.74	0.82513	0.338
	Treatment	35	3.45714	4.154	0.70215	
	Control	34	4.91176	4.6343	0.79478	
DMFT	Patient	23	1.73913	2.4162	0.50381	0.124
	Treatment	29	0.82759	1.3646	0.2534	
	Control	32	0.4375	0.9483	0.16763	
DMFS	Patient	23	2.26087	3.2783	0.68357	0.145
	Treatment	29	1	1.5584	0.28939	
	Control	32	0.625	1.4756	0.26085	
Plaque index	Patient	40	0.145	0.2025	0.03202	0.176
	Treatment	40	0.14185	0.1616	0.02555	
	Control	40	0.0868	0.0984	0.01555	
Gingival index	Patient	40	0.00432	0.0162	0.00256	0.134
	Treatment	40	0.02732	0.069	0.01091	
	Control	40	0.02113	0.05	0.00791	

Kruskal Wallis Test.

**Table 5:** Assessment of halitosis.

	Patient group (*N* = 40)	Treatment group (*N* = 40)	Control group (*N* = 40)	*p* value
Organoleptic measurement				
No or barely noticeable odor	0.0 (0.0%)	21.0 (52.5%)	24.0 (60.0%)	<0.001
Weak but clearly noticeable odor	27.0 (67.5%)	8.0 (20.0%)	8.0 (20.0%)	
Moderate or strong odor	13.0 (32.5%)	11.0 (27.5%)	8.0 (20.0%)	
Parental perception of odor in the child				
Yes	39.0 (97.5%)	19.0 (47.5%)	14.0 (35.0%)	<0.001
No	1.0 (2.5%)	21.0 (52.5%)	26.0 (65.0%)	

Pearson's Chi-squared test.

**Table 6:** Assessment of oral bad habits.

	Patient group (*N* = 40)	Treatment group (*N* = 40)	Control group (*N* = 40)	*p* value
Tongue thrusting	3.0 (7.5%)	0.0 (0.0%)	0.0 (0.0%)	0.0461
Lip sucking	3.0 (7.5%)	1.0 (2.5%)	1.0 (2.5%)	0.4341
Lip biting	2.0 (5.0%)	1.0 (2.5%)	0.0 (0.0%)	0.3591
Nail biting	5.0 (12.5%)	2.0 (5.0%)	2.0 (5.0%)	0.3391
Bruxism	9.0 (22.5%)	5.0 (12.5%)	2.0 (5.0%)	0.0691

Pearson's Chi-squared test.

**Table 7:** Assessment of dentofacial findings.

	Patient group (*N* = 40)	Treatment group (*N* = 40)	Control group (*N* = 40)	*p* value
V-shaped narrowing in the maxillary arch	38.0 (95.0%)	38.0 (95.0%)	0.0 (0.0%)	<0.001
Adenoid face	38.0 (95.0%)	30.0 (75.0%)	0.0 (0.0%)	<0.001
Teeth crowding	12.0 (30.0%)	17.0 (42.5%)	7.0 (17.5%)	0.051
Macroglossia	26.0 (65.0%)	27.0 (67.5%)	5.0 (12.5%)	<0.001
Open mouth posture	33.0 (82.5%)	27.0 (67.5%)	4.0 (10.0%)	<0.001
Anterior and lower tongue position	26.0 (65.0%)	25.0 (62.5%)	0.0 (0.0%)	<0.001
Dry, chapped lips	37.0 (92.5%)	7.0 (17.5%)	1.0 (2.5%)	<0.001
Position of mandibula				
Normal	0.0 (0.0%)	9.0 (22.5%)	35.0 (87.5%)	<0.001
Prognatic	3.0 (7.5%)	1.0 (2.5%)	4.0 (10.0%)	
Retrognatic	37.0 (92.5%)	30.0 (75.0%)	1.0 (2.5%)	
Position of maxillary anterior teeth				
Normal	25.0 (62.5%)	22.0 (55.0%)	37.0 (92.5%)	<0.001
Prominent	15.0 (37.5%)	18.0 (45.0%)	3.0 (7.5%)	
Position of mandibular anterior teeth				
Normal	20.0 (50.0%)	30.0 (75.0%)	39.0 (97.5%)	<0.001
Retrognatic	20.0 (50.0%)	10.0 (25.0%)	1.0 (2.5%)	
Dentition period				
Primary dentition	16.0 (40.0%)	10.0 (25.0%)	6.0 (15.0%)	0.111
Mixed dentition	17.0 (42.5%)	23.0 (57.5%)	28.0 (70.0%)	
Permanent dentition	7.0 (17.5%)	7.0 (17.5%)	6.0 (15.0%)	

Pearson's Chi-squared test.

**Table 8:** Assessment of interarch dental occlusal relationship.

	Patient group (*N* = 40)	Treatment group (*N* = 40)	Control group (*N* = 40)	*p* value
Sagittal relationship				
Normal occlusion	6.0 (21.4%)	9.0 (30.0%)	20.0 (58.8%)	<0.001
Class I malocclusion	4.0 (14.3%)	6.0 (20.0%)	10.0 (29.4%)	
Class II division 1 malocclusion	18.0 (64.3%)	15.0 (50.0%)	4.0 (11.8%)	
Transversal relationship				
Posterior crossbite	9.0 (22.5%)	10.0 (25.0%)	5.0 (12.5%)	0.335
Normal, no crossbite	31.0 (77.5%)	30.0 (75.0%)	35.0 (87.5%)	
Vertical relationship				
Normal overbite	20.0 (50.0%)	18.0 (45.0%)	23.0 (57.5%)	0.244
Anterior open bite	8.0 (20.0%)	11.0 (27.5%)	3.0 (7.5%)	
Deep bite	12.0 (30.0%)	11.0 (27.5%)	14.0 (35.0%)	

Pearson's Chi-squared test.

**Table 9:** Assessment of OHRQoL-ECOHIS scale.

	Group	Mean	SD	SE	*p*
Child impact section					
Child symptoms	Patient	0.95	1.319	0.209	0.984
	Treatment	1.03	1.387	0.219	
	Control	1.05	1.518	0.240	
	Total	1.01	1.399	0.128	
Child function	Patient	2.15	3.363	0.532	0.816
	Treatment	2.65	3.634	0.575	
	Control	2.45	3.404	0.538	
	Total	2.42	3.446	0.315	
Child psychology	Patient	0.48	1.261	0.199	0.936
	Treatment	0.60	1.516	0.240	
	Control	0.53	1.519	0.240	
	Total	0.53	1.426	0.130	
Child self-image and social interaction	Patient	1.90	1.809	0.286	0.049^∗^
	Treatment	1.48	2	0.316	
	Control	0.98	1.459	0.231	
	Total	1.45	1.796	0.164	
Total child score	Patient	5.48	6.869	1.086	0.519
	Treatment	5.75	7.2	1.138	
	Control	5	6.887	1.089	
	Total	5.41	6.935	0.633	
Family impact section					
Parental distress	Patient	0.65	1.494	0.236	0.494
	Treatment	1.23	2.270	0.359	
	Control	1.13	2.09	0.330	
	Total	1	1.979	0.181	
Family function	Patient	0.30	0.883	0.140	0.255
	Treatment	0.55	1.154	0.182	
	Control	0.73	1.502	0.237	
	Total	0.53	1.209	0.110	
Total parent score	Patient	0.95	2.309	0.365	0.402
	Treatment	1.78	3.363	0.532	
	Control	1.85	3.446	0.545	
	Total	1.53	3.084	0.282	
Total ECOHIS score	Patient	6.43	8.918	1.410	0.690
	Treatment	7.53	10.163	1.607	
	Control	6.85	9.991	1.580	
	Total	6.93	9.635	0.880	

Kruskal Wallis Test.

## Data Availability

The data used to support the findings of this study are available from the corresponding author is upon request.
